# An NBD Derivative of the Selective Rat Toxicant Norbormide as a New Probe for Living Cell Imaging

**DOI:** 10.3389/fphar.2016.00315

**Published:** 2016-09-23

**Authors:** Claudio D'Amore, Genny Orso, Fabio Fusi, Mario A. Pagano, Giovanni Miotto, Alessia Forgiarini, Sara De Martin, Giulia Castellani, Giovanni Ribaudo, David Rennison, Margaret A. Brimble, Brian Hopkins, Alessandro Ferrarese, Sergio Bova

**Affiliations:** ^1^Department of Surgical and Biomedical Sciences, University of PerugiaPerugia, Italy; ^2^IRCCS “E. Medea”Conegliano, Italy; ^3^Department of Life Sciences, University of SienaSiena, Italy; ^4^Department of Pharmaceutical and Pharmacological Sciences, University of PaduaPadua, Italy; ^5^Department of Molecular Medicine, University of PaduaPadua, Italy; ^6^Istituto di Ricerca Pediatrica Città della SperanzaPadua, Italy; ^7^School of Chemical Sciences, University of AucklandAuckland, New Zealand; ^8^Landcare ResearchLincoln, New Zealand

**Keywords:** norbormide, ER tracker, fluorescent probes, sulphonylurea receptors, LX2 cells

## Abstract

Norbormide (NRB) is a unique compound that acts directly on rat vascular myocytes to trigger a contractile process, through an as yet unknown mechanism, which results in the selective contraction of rat peripheral arteries. To gain insight into the mechanisms involved in NRB rat-selective activity, we investigated the subcellular distribution of NRB-AF12, a nitrobenzoxadiazole (NBD)-derivative of NRB, in living NRB-sensitive and NRB-insensitive cells. In both cell types, NRB-AF12 localized to the endoplasmic reticulum (ER), Golgi apparatus, mitochondria, lysosomes, and endosomes; however, in NRB-sensitive cells, the fluorescence also extended to the plasma membrane. NRB-AF12 was rapidly internalized into the cells, could easily be washed out and then reloaded back into the same cells, all with a high degree of reproducibility. Cells exposed for 24 h to NRB-AF12 did not show apparent signs of toxicity, even at concentrations of the dye (10 μM) much higher than those required for fluorescence labeling (500 ηM). The distribution pattern of NRB-AF12 fluorescence was near identical to that of ER-Tracker® (Er-Tr), a fluorescent derivative of glibenclamide, a known K_ATP_ channel blocker. Displacement tests did not demonstrate, but at the same time did not rule out the possibility of a common target for ER-Tr, NRB-AF12, NRB, and glibenclamide. On the basis of these results we hypothesize a common target site for NRB-AF12 and ER-Tr, and a similar target profile for NRB and glibenclamide, and propose NRB-AF12 as an alternative fluorescence probe to ER-Tracker. Furthermore, NRB-based fluorescence derivatives could be designed to selectively label single cellular structures.

## Introduction

**Figure 1:**
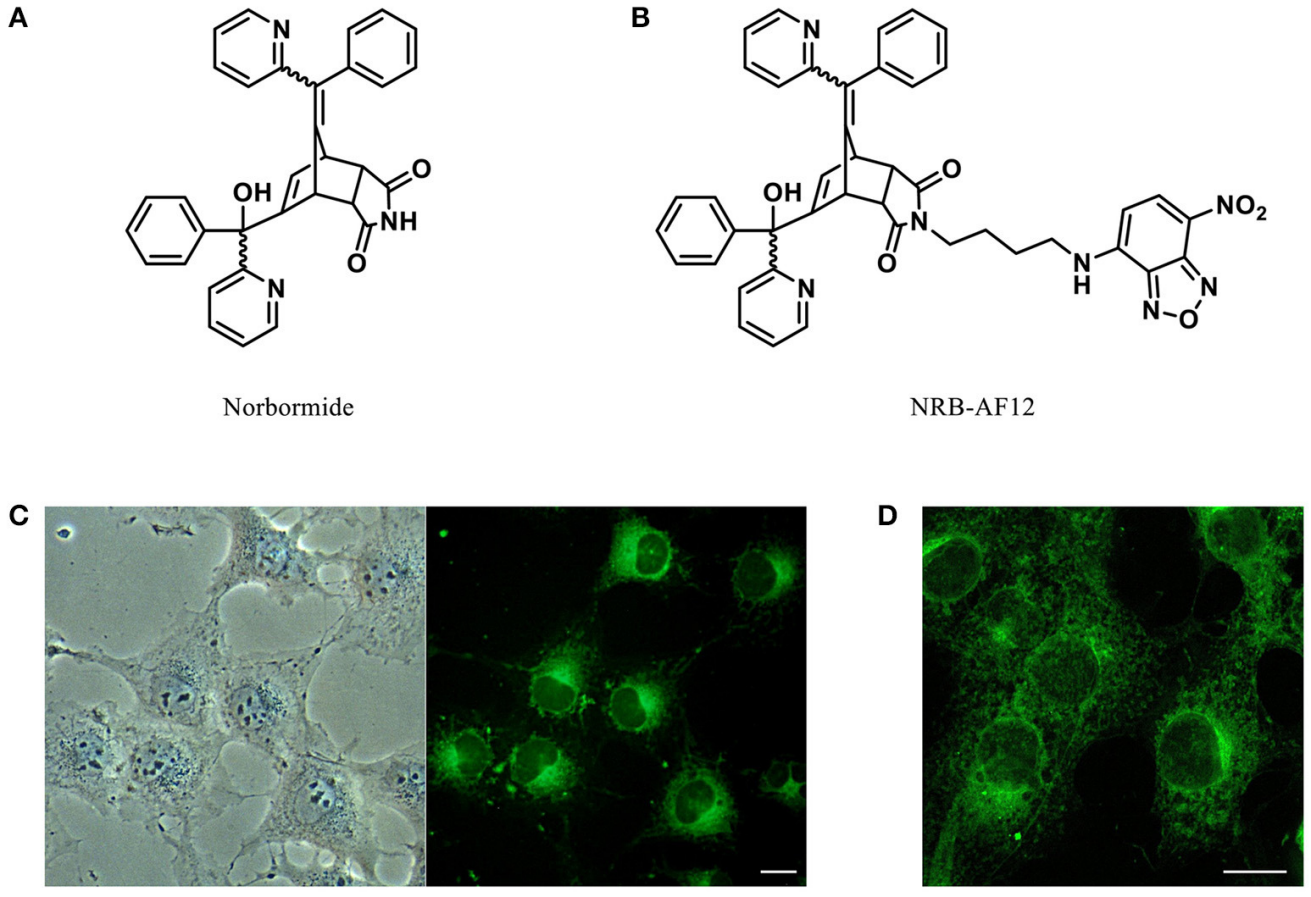
**Chemical structures of (A) Norbormide and (B) its fluorescent derivative NRB-AF12**. **(C)** Live cell imaging of LX2 hepatic stellate cells fluorescently labeled with 500 ηM NRB-AF12 for 30 min. Magnification 40x; scale bar 20 μm. **(D)** LX2 cells stained with NRB-AF12 were fixed and imaged by confocal microscopy. Magnification 60x; scale bar 20 μm.

Norbormide (NRB, see Figure 1A for structure) is a unique vasoactive compound endowed with species-specific vasoconstrictor activity that targets the peripheral blood vessels of the rat (Clarke, 1965; Roszkowski, 1965; Bova et al., 1996). It also provokes a vasorelaxant effect on arteries of several non-rat species, as well as on rat large-caliber vessels (Roszkowski, 1965; Poos et al., 1966; Cavalli et al., 2004). NRB is a mixture of up to eight stereoisomers, four *endo*-isomers and four *exo*-isomers; among them, only the *endo*-isomers demonstrate vasocontractile and rat toxicant activities (Poos et al., 1966), whereas both *endo*- and *exo*-isomers have vasodilatatory activity in rat aorta and in non-rat arteries (Cavalli et al., 2004). The contractile effect of NRB on rat peripheral blood vessels is endothelium-independent, and is of myogenic nature as confirmed by its action on single rat caudal artery myocytes (Fusi et al., 2002; Cavalli et al., 2004). NRB binding sites and the molecular mechanisms involved in its biological effects in vascular tissue have yet to be fully characterized; it has been proposed that NRB-induced vasoconstriction may result from an interaction with a phospholipase C-coupled receptor (Bova et al., 2001), whereas its relaxant effect may be caused by an inhibitory action on plasma membrane voltage-dependent L-type Ca^2+^ channels (Fusi et al., 2002). NRB has also been shown to activate the mitochondrial permeability transition pore (mPTP) in isolated rat mitochondria, but not in mitochondria obtained from either guinea-pigs or mice. This suggests that the effect on the mPTP, like NRB-vasoconstriction, is also species-specific. However, since this effect could also be induced in mitochondria isolated from different organs of the rat (Ricchelli et al., 2005; Zulian et al., 2007, 2011), unlike vasoconstriction, mPTP activation is not tissue-specific. It is therefore unclear whether NRB-induced activation of the mPTP constitutes a role in the vasoconstrictor effect of NRB.

Correspondingly, addressing the localization of NRB could help characterize the molecular mechanisms underlying the species- and tissue-selective actions of NRB; the original primary objective of this study was therefore to develop a fluorescent derivative of NRB (NRB-AF12, see Figure 1B for structure) in order to compare its subcellular distribution in NRB-sensitive and NRB-insensitive cells. However, since preliminary results indicated a strong overlapping fluorescent distribution between NRB-AF12 and that of ER-Tracker® (ER-Tr), a commercially available fluorescent probe widely used as a selective marker for endoplasmic reticulum (ER), we extended our goals to include a comparison of the staining profiles of the two dyes. The results indicate that NRB-AF12 and ER-Tr show a near identical pattern of fluorescence distribution in both NRB-sensitive and NRB-insensitive cells, and that this fluorescence profile differs between the two cell types. Furthermore, we found that both dyes label not only ER (as would be expected with ER-Tr), but also Golgi apparatus and mitochondria. In addition, unlike ER-Tr, NRB-AF12 was also able to label lysosomes and endosomes.

## Materials and methods

### Chemistry

To a solution of *endo*-NRB (1.0 g, 2.0 mmol) in DMF (6 ml) was added NaH (160 mg, 4.0 mmol, 60% w/w in oil), and the resulting mixture stirred at room temperature for 15 min, then at 80°C for a further 15 min A solution of 4-[(*tert*-butoxycarbonyl)amino]butyl methanesulfonate (Cao et al., 2016) (0.59 g, 2.2 mmol) in DMF (2 ml) was added, and the resulting mixture stirred at 100°C for 4 h. The reaction mixture was then allowed to cool, diluted with water and extracted with EtOAc. The separated organic phase was washed with brine, dried over anhydrous Na_2_SO_4_, filtered and the solvent removed *in vacuo*. Purification by column chromatography (hexane/EtOAc, 1:1) afforded *N*-4′-[(*tert*-butoxycarbonyl)amino] butyl-5-(α-hydroxy-α-2-pyridylbenzyl)-7-(α-2-pyridylbenzylidene)-5-norbornene-2,3-dicarboximide as an *endo*-exclusive mixture of four stereoisomers (white solid; 1.09 g, 1.60 mmol, 80%). ^1^H NMR (400 MHz, CDCl_3_) δ 1.30-1.68 (13H, m, C(CH_3_)_3_ and NCH_2_CH_2_CH_2_CH_2_NH), 3.05-3.20 (2H, m, NCH_2_CH_2_CH_2_CH_2_NH), 3.24-3.70 (4.2H, m, H-2, H-3, W/H-4 and NCH_2_CH_2_CH_2_CH_2_NH), 3.85–3.90 (0.4H, m, U/H-1 and V/H-1), 3.91-3.95 (0.4H, m, Y/H-4), 4.10–4.13 (0.1H, m, U/H-4), 4.26–4.30 (0.3H, m, V/H-4), 4.42–4.48 (0.6H, m, W/H-1 and Y/H-1), 5.55–5.67 (1.7H, m, OH and V/H-6 and Y/H-6), 6.02–6.04 (0.1H, m, U/H-6), 6.05–6.07 (0.2H, m, W/H-6), 6.70–7.62 (16H, m, ArH), 8.66–8.39 (2H, m, αPyr). To a solution of *N*-4′-[(*tert*-butoxycarbonyl)amino]butyl-5-(α-hydroxy-α-2-pyridylbenzyl)-7-(α-2-pyridylbenzylidene)-5-norbornene-2,3-dicarboximide (0.34 g, 0.5 mmol) in DCM (10 ml) was added TFA (2 ml), and the mixture stirred at room temperature for 3 h. The solvent was then removed *in vacuo* to afford *N*-4′-aminobutyl-5-(α-hydroxy-α-2-pyridylbenzyl)-7-(α-2-pyridylbenzylidene)-5-norbornene-2,3-dicarboximide trifluoroacetate as an *endo*-exclusive mixture of four stereoisomers [colorless gum (quant.)], which was used without further purification. To a solution of *N*-4′-aminobutyl-5-(α-hydroxy-α-2-pyridylbenzyl)-7-(α-2-pyridylbenzylidene)-5-norbornene-2,3-dicarboximide trifluoroacetate (100 mg, 0.14 mmol) in DCM (4 ml) was added DIPEA (73 μl, 0.42 mmol), and the mixture stirred at room temperature for 30 min. NBD-Cl (4-chloro-7-nitrobenzofurazan) (28 mg, 0.14 mmol) was added and the resulting mixture stirred at room temperature for a further 6 h. The mixture was then diluted with DCM, washed with saturated aq. NaHCO_3_, then brine, and the separated organic phase dried over anhydrous Na_2_SO_4_, filtered and the solvent removed *in vacuo*. Purification by column chromatography (DCM) afforded 5-(α-hydroxy-α-2-pyridylbenzyl)-*N*-4′-[(7″-nitro-2″,1″,3″-benzoxadiazol-4”-yl)amino]butyl-7- (α-2- pyridylbenzylidene)-5-norbornene-2,3-dicarboximide (NRB-AF12) as an *endo*-exclusive mixture of four stereoisomers (orange solid; 50 mg, 0.067 mmol, 48%). M.p. 108-112°C; ^1^H NMR (400 MHz, CDCl_3_/MeOD 50:1 v/v) δ 1.61–1.84 (4H, m, NCH_2_CH_2_CH_2_CH_2_N), 3.34–3.73 (6.2H, m, NCH_2_CH_2_CH_2_CH_2_N, H-2, H-3 and W/H-4), 3.89–3.98 (0.7H, m, V/H-1 and Y/H-4), 3.99–4.02 (0.1H, m, U/H-1), 4.18–4.24 (0.4H, m, V/H-4 and U/H-4), 4.28–4.38 (0.6H, m, Y/H-1 and W/H-1), 5.56–5.62 (0.7H, m, V/H-6 and Y/H-6), 6.07–6.09 (0.1H, m, U/H-6), 6.12–6.14 (0.2H, m, W/H-6), 6.14–6.24 (1H, m, H-5″), 6.79–7.75 (16H, m, ArH), 8.37–8.65 (3H, m, αPyr and H-6″).

### Fluorescence spectra of NRB-AF12

Fluorescence spectra of NRB-AF 12 were obtained by dissolving the dye in water (for details on the methodology see Figure S1 in the Supplementary Material)

### Cell culture

LX2 (a human immortalized hepatic stellate cell line), HSC-T6 (a rat immortalized hepatic stellate cell line), and HeLa (a human cervical carcinoma cell line) cells were cultured at 37°C in an atmosphere of 5% CO_2_ in Dulbecco's Modified Eagle Medium (Euroclone - Milan, Italy) supplemented with 10% Fetal Bovine Serum (Euroclone), 2 mM L-Glutamine and antibiotics. HepG2 cells (a human hepatocarcinoma cell line) were cultured at 37°C in an atmosphere of 5% CO_2_ in Eagle's Minimum Essential Medium (Euroclone) containing 10% Fetal Bovine Serum, 2 mM L-Glutamine and antibiotics.

### VSMC isolation

Smooth muscle cells were freshly isolated from the rat tail main artery under the following conditions: a 5-mm long piece of artery was incubated at 37°C for 40–45 min in 2 ml of 0.1 mM Ca^2+^ external solution containing 20 mM taurine, 1.35 mg/ml collagenase (type XI), 1 mg/ml soybean trypsin inhibitor, and 1 mg/ml BSA, which was lightly bubbled with a 95% O_2_–5% CO_2_ gas mixture to gently agitate the enzyme solution. Cells were stored in 0.05 mM Ca^2+^ external solution containing 20 mM taurine and 0.5 mg/ml BSA at 4°C under normal atmosphere, and were used for experiments within 2 days following isolation (Fusi et al., 2016).

### Live-cell imaging

All cell lines tested were stained immediately before imaging. Briefly, cells were trypsinized, counted and seeded in glass bottom μ-Dishes (Ibidi - Munich, Germany) at 2 × 10^4^ cells/dish, and incubated for 24 h at 37°C in an atmosphere of 5% CO_2_. Cells were rinsed with Dulbecco's Modified Eagle Medium without phenol red (Euroclone) and loaded with 500 ηM NRB-AF12, 500 ηM ER-Tr red or green (#E34250 and #E34251; Thermo Fisher), and 500 ηM pHrodo red (#P10361; Thermo Fisher) at 10 μg/ml. In another experiment, LX2 cells were seeded in glass bottom μ-Dishes at 2 × 10^4^ cells/dish and incubated for 24 h at 37°C in an atmosphere of 5% CO_2_. Cells were transfected with either 500 ηg of MitoDS-red or KDEL-red vector using Lipofectamine LTX Reagent (Invitrogen), according to the manufacturer specifications. At 24-h post-transfection, cells were stained with either 500 ηM NRB-AF12 or 500 ηM ER-Tr green. Cells were mounted in a Okolab microscope stage heated chamber warmed to 37°C. All images were collected with a Yokogawa CSU-X1 spinning disk confocal on a Nikon Ti-E inverted microscope equipped with a Plan Apo 60x NA 1.4 objective. Probes were excited with a 488 and 561 nm Lasers. To separate the individual emissions a dichroic filter 405/488/561/635-25 (Semrock, Rochester, NY) followed by a 440-40/521-21/607-34/700-45 filter (Semrock, Rochester, NY) were used and subsequently filtered further using band-pass filters (Semrock BP-525/30-25 and BP-607/36-25 for the green and red channels, respectively). Images were acquired with an Andor Technology iXon3 DU-897-BV EMCCD camera. For time-lapse experiments, images were collected every 10 s, using an exposure time of 500 ms.

### Immunocytochemistry

To evaluate cell retention of the probe post-fixation/permeabilization, LX2 cells (2 × 10^4^) were plated on a 12 mm glass coverslip placed in a well of a 24 well-plate and incubated for 24 h at 37°C in an atmosphere of 5% CO_2_. The cells were then treated with 500 ηM NRB-AF12 for 30 min, fixed with 4% paraformaldehyde for 15 min at room temperature and immediately mounted on microscope slides, or fixed with 4% paraformaldehyde, permeabilized with either 0.1% Triton X-100 (10 min at room temperature) or ice cold methanol (5 min at −20°C), and subsequently mounted.

In order to evaluate NRB-AF12 co-localization with endoplasmic reticulum, Golgi apparatus and with the sulfonylurea receptor (SUR) subunits of the ATP-sensitive potassium channels, cells were fixed with 4% paraformaldehyde for 15 min at room temperature, and permeabilized with ice cold methanol for 5 min at −20°C. ER was highlighted with antibodies directed against the ER resident protein calreticulin (1:200; Abcam #ab2907); Golgi apparatus was highlighted with antibodies directed against the Golgi apparatus structural protein GM130 (1:500; BD Biomedsciences #610822); SUR2 subunits of K_ATP_ channel were highlighted using either anti-SUR2A (1:750; Abcam #ab174629) or anti-SUR2B (1:750; Abcam #ab174631) antibodies. All primary antibodies were incubated for 90 min at room temperature. Following a PBS wash, cells were incubated with Cy™3 goat anti-rabbit IgG fluorescent secondary antibody (1:500; Jackson ImmunoResearch #111-165-003) or with Cy™3 goat anti mouse IgG fluorescent secondary antibody (1:500; KPL #072-01-18-06) for 60 min at room temperature in the presence of either 500 ηM NRB-AF12 or 500 ηM ER-Tr green. Finally, coverslips were mounted on microscope slides using Mowiol 40–88 (Sigma Aldrich). Confocal images were acquired with a Laser Scanning Confocal Microscope (LSCM) D-Eclipse C1 SHV Nikon system equipped with a Nikon Eclipse E600 microscope and 3 laser diode modules (488/543/637 nm), using a CFI Plan Apochromat 60x NA 1.4 objective and analyzed using NIS Elements software (Nikon) and the open-source platform for biological-image analysis Fiji software.

### Drosophila melanogaster

Drosophila strain W[1118], stock number 5905 was obtained from Bloomington stock center (Indiana University, USA) and used to perform live imaging experiments. Fly larvae having reached the third instar stage whilst feeding on standard food were dissected in hemolymph-like HL3 saline (70 mM NaCl, 5 mM KCl, 1.5 mM CaCl_2_, 20 mM MgCl_2_, 10 mM NaHCO_3_, 5 mM trehalose, 115 mM sucrose, 5 mM sodium HEPES, pH 7.2). For live tissue imaging ER-Tr red and NRB-AF12 were added to the medium of dissected larvae and images acquired using a Nikon eclipse C1 confocal microscope and a Nikon Fluor 60x NA 1.00 water objective.

### Biocompatibility

1 × 10^5^ LX2 cells, 1 × 10^5^ HepG2 cells and 5 × 10^4^ HSC-T6 cells were plated in 12 well-plates in complete culture medium and incubated for 24 h at 37°C. Cells were then treated with increasing doses (0.1, 1 and 10 μM) of NRB, NRB-AF12, glibenclamide and ER-Tr for 24 h. Cell viability was assessed by the Trypan Blue exclusion method. Briefly, cells were trypsinized, resuspended in complete medium, diluted 1:2 with a 0.4% Trypan Blue solution and finally counted using a hemacytometer under a light-microscope.

### Hazards related to the use of NRB and its derivatives for research purposes

In HSNO (Hazardous Substances and New Organisms) terms, a substance is considered hazardous if it triggers any one of the threshold levels for any of the hazardous properties listed in the USA Environmental Protection Agency (EPA) guidelines, one of which is toxicity. NRB is extremely toxic to rats and as such is classified as an “extremely hazardous chemical” in the US and elsewhere. However, one of the key features of NRB is that its toxic activity is uniquely limited to rats, having little or no activity in any other species tested (including humans). In fact NRB's lethal activity is even further restricted as it seems only to be toxic to species within the Rattus genus, with other rat genera being little or not affected. As a consequence, the NRB and the NRB derivatives reported in this research paper poses little or no hazard to the researchers, and as such no special conditions or safety precautions are required to work with these substances other than those required to conform to standard laboratory practices.

### Statistical analysis

All values are expressed as means ± standard error (SE) of n observations/group. Analysis of co-localization was performed using Pearson's correlation coefficient. Comparisons of more than two groups were made with a one-way ANOVA using *post-hoc* Tukey's test. Comparison of two groups was obtained by the Student's *t*-test for unpaired data when appropriate. Differences were considered statistically significant at values of *P* < 0.05.

## Results

The fluorescence spectra of NRB-AF12 are shown in Figure S1 in Supplementary Material.

The distribution of NRB-AF12 was initially evaluated in NRB-insensitive cells.

**Figure 2:**
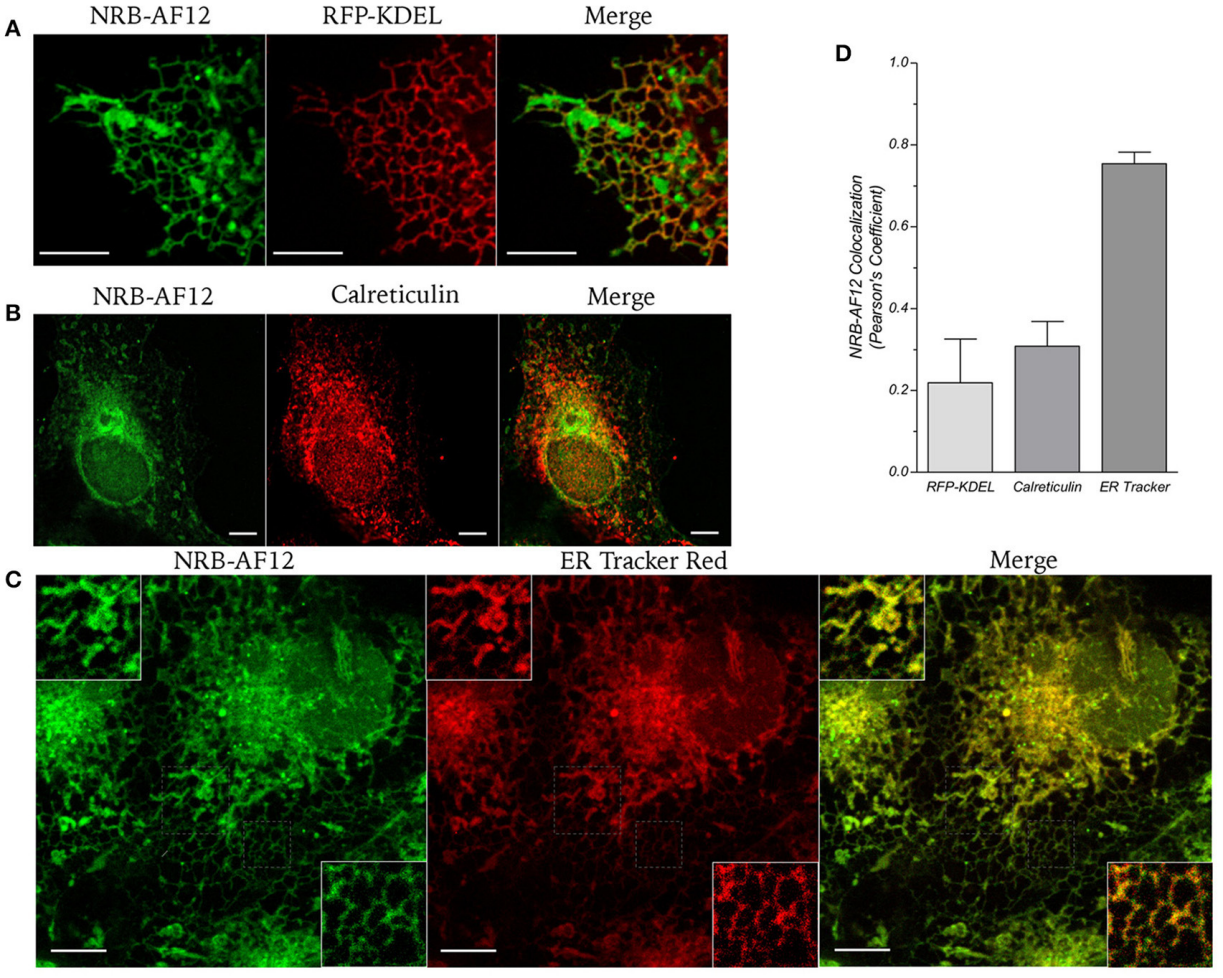
**(A)** Live cell imaging of LX2 cells transfected with ER-reporter RFP-KDEL plasmid and subsequently stained with NRB-AF12. **(B)** Immunostaining of LX2 cells for the endoplasmic reticulum marker calreticulin; cells were counter-labeled with NRB-AF12. **(C)** Confocal live cell imaging of LX2 cells fluorescently labeled with 500 ηM NRB-AF12 and 500 ηM ER-Tr red. Insets show magnification of the pictures. Magnification 60x; scale bar 10 μm. **(D)** Quantification of NRB-AF12 co-localization with RFP-KDEL, Calreticulin and ER-Tr is shown as Pearson's coefficient. (*n* ≥ 10).

**Figure 3:**
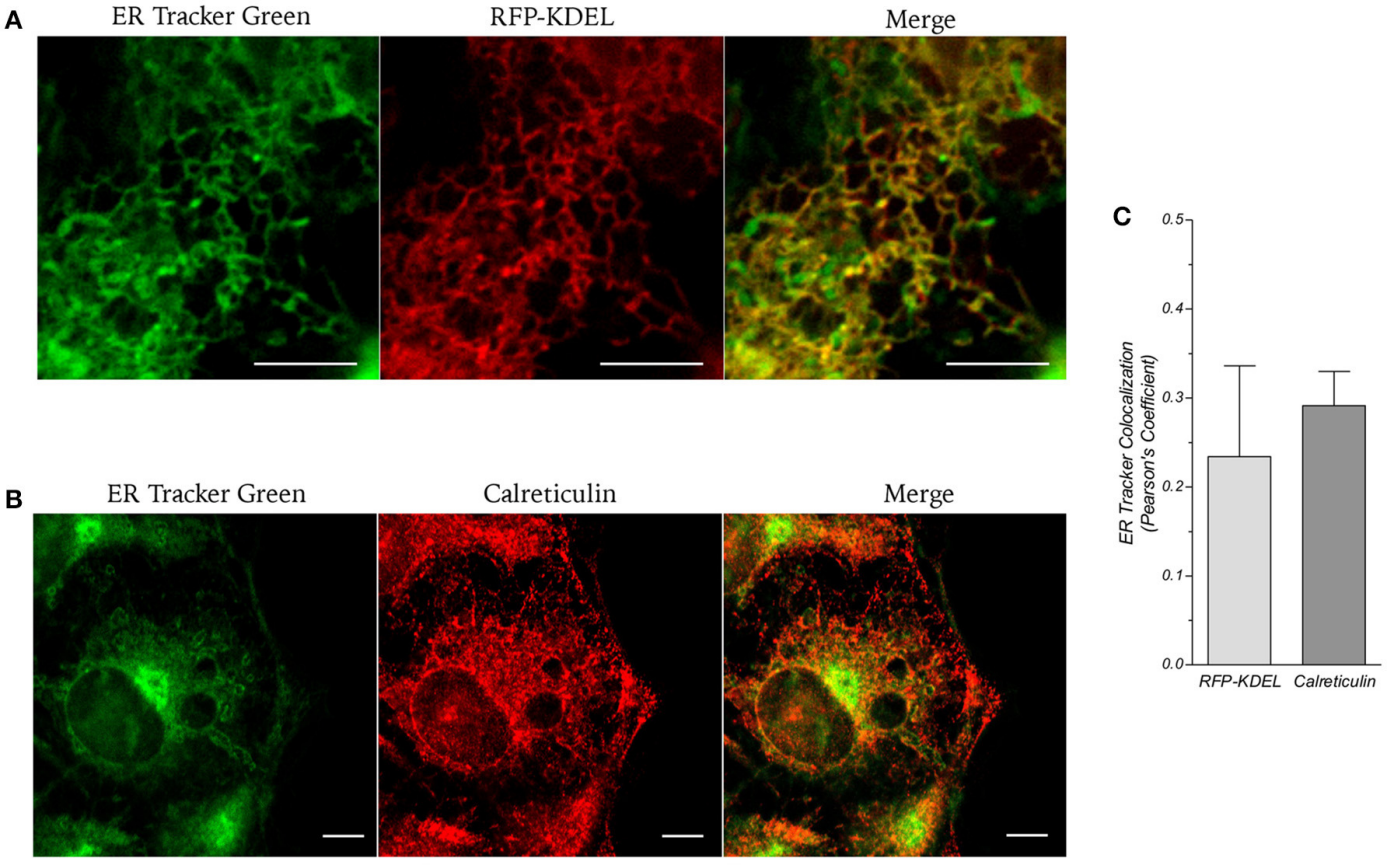
**(A)** Live cell imaging of LX2 cells transfected with ER-reporter RFP-KDEL plasmid and subsequently stained with ER-Tr. **(B)** Immunostaining of LX2 cells for the endoplasmic reticulum marker calreticulin; cells were counter-labeled with NRB-AF12. Magnification 60x; scale bar 10 μm. **(C)** Quantification of ER-Tr co-localization with RFP-KDEL and Calreticulin is shown as Pearson's coefficient (*n* ≥ 10).

Figure 1C shows phase contrast microscopy images and conventional epifluorescence microscopy images of living LX2 cells incubated with 500 ηM NRB-AF12. The images clearly show a meshwork distribution of the fluorescence with a sharp demarcation of the nuclei and an accumulation in perinuclear areas; moreover, NRB-AF12 did not stain either the nuclei or the plasma membrane. NRB-AF12 internalization into the cell was fast, commencing after only a few seconds and reaching completion within a few minutes, following its addition to the incubation medium. A similar fluorescence pattern was also observed in fixed LX2 cells (Figure 1D), however, the NRB-AF12 signal was not retained post-permeabilization (data not shown). The sub-cellular distribution of NRB-AF12 was investigated in more detail in LX2 cells using confocal microscopy in combination with fluorescent probes and/or antibodies targeted to specific subcellular structures. To evaluate the distribution of NRB-AF12 in the ER, LX2 cells were transfected with the ER-reporter red fluorescent protein (RFP)-KDEL plasmid and subsequently loaded with the dye. The results, reported in Figures 2A,D, show that the NRB-AF12 signal significantly overlapped with that of (RFP)-KDEL (Pearson's Coefficient: 0.21 ± 0.10; *n* ≥ 10), but a larger staining distribution of NRB-AF12 was observed which indicated other targets in addition to ER. Similar results were observed in LX2 fixed cells labeled with NRB-AF12 and immunostained for the ER resident protein Calreticulin (Pearson's Coefficient: 0.3 ± 0.06; *n* ≥ 10) (Figures 2B–D). We also compared the staining pattern of NRB-AF12 with that of ER-Tr, a commercial fluorescent probe widely used to label ER. Surprisingly, co-labeling of LX2 cells with NRB-AF12 and ER-Tr resulted in a strong overlap between fluorescent dyes (Figure 2C), confirmed by the high rate of co-localization index (Pearson's coefficient: 0.75 ± 0.02; *n* ≥ 15). This remarkable similarity between the two dyes was further supported by the observation that ER-Tr, as well as NRB-AF12, were not selective for the ER, as demonstrated by co-staining LX2 cells with ER-Tr plus either RFP-KDEL (Figure 3A) or Calreticulin (Figure 3B) (Pearson's coefficient: 0.23 ± 0.1 and 0.29 ± 0.03, respectively for RFP-KDEL and Calreticulin; *n* ≥ 10; Figure 3C).

**Figure 4:**
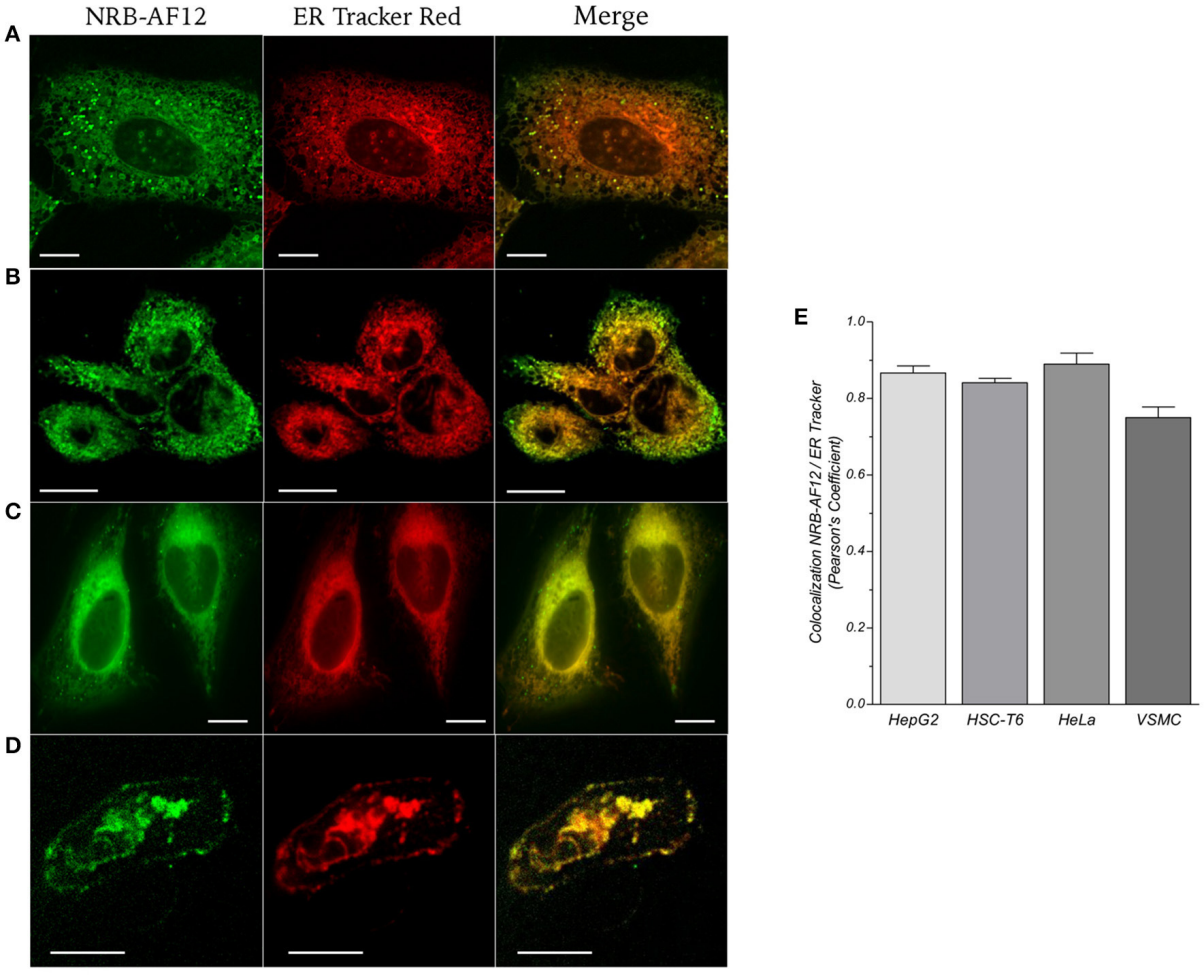
**Representative live cells microphotographs showing co-localization between NRB-AF12 (left column) and ER-Tr red (mid column) in (A) HepG2, (B) HSC-T6, (C) HeLa, and (D) primary rat vascular smooth muscle cells (VSMC) from caudal artery**. For each panel, merged fluorescence is shown in right column. Magnification 60x; scale bar, 10 μm. **(E)** Quantification of NRB-AF12 and ER-Tr co-localization is shown as Pearson's coefficient (n ≥ 15).

The NRB-AF12 and ER-Tr subcellular distribution patterns and co-localization rates were confirmed using other NRB-insensitive cells (i.e., HepG2, HSC-T6, and HeLa cells; Figures 4A–C), and using NRB-sensitive cells such as freshly isolated rat caudal artery myocytes (Figure 4D). Interestingly, in this latter cell type, both fluorescent compounds stained not only intracellular structures, but also the plasma membrane. Co-localization rates of NRB-AF12 and ER-Tr in HepG2, HSC-T6, HeLa and primary rat vascular smooth muscle cells is reported in Figure 4E as Pearson's coefficient. These results indicates that NRB-AF12 labels intracellular structures with a pattern of distribution that is apparently very similar to that of ER-Tr, and is not confined to ER.

We then investigated, using LX2 cells, which non-ER subcellular organelles were labeled by NRB-AF12, and compared the results with those obtained with ER-Tr, focusing our attention on the Golgi apparatus, mitochondria, lysosomes, and endosomes.

**Figure 5:**
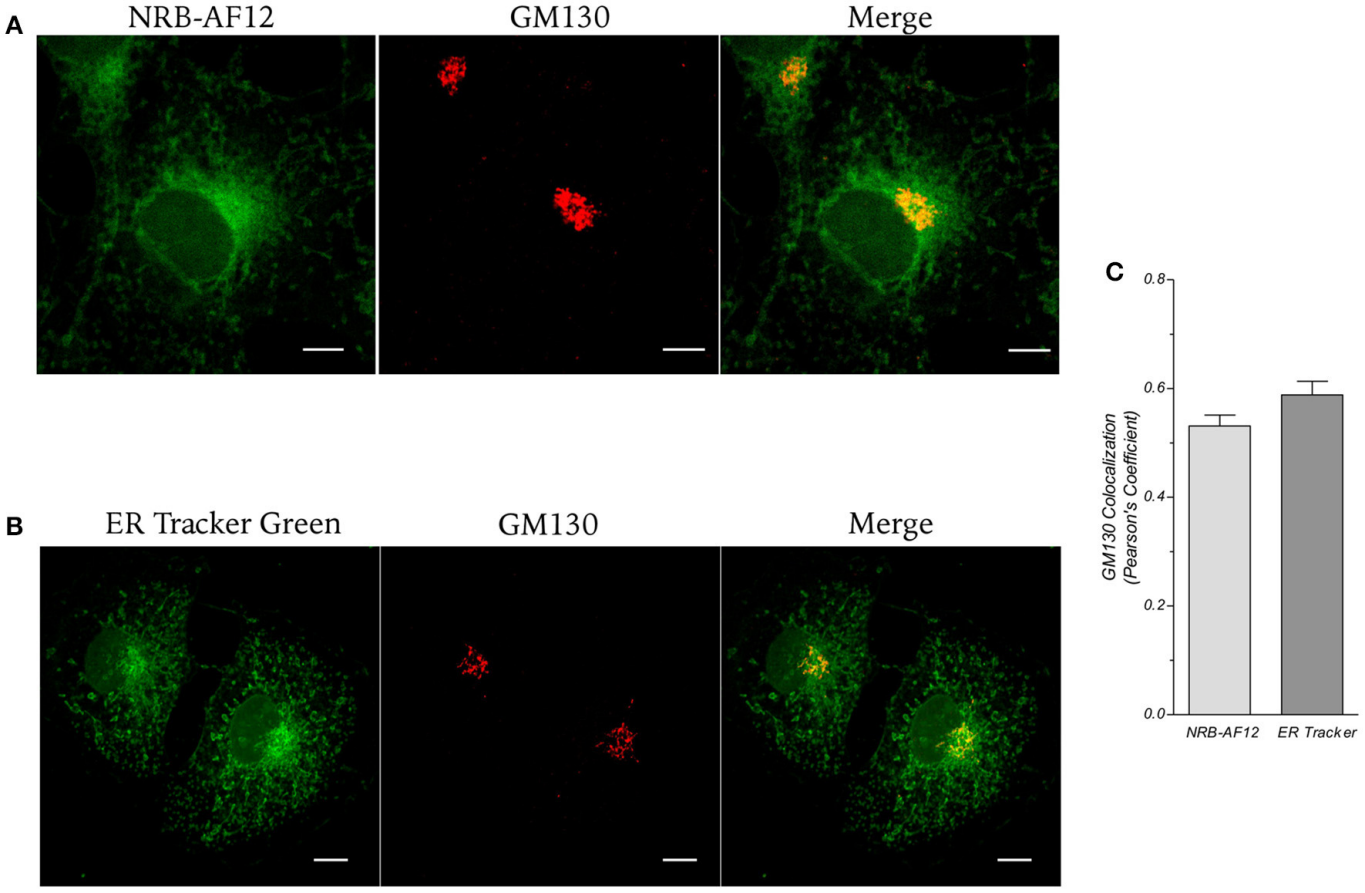
**LX2 fixed cells immunostained for the Golgi apparatus structural protein GM130; cells were counter-labeled with (A) NRB-AF12 or (B) ER-Tr green**. Magnification 60x; scale bar 10 μm. **(C)** Co-localization measure is shown as Pearson's coefficient (*n* ≥ 10).

The Golgi apparatus was investigated by co-staining fixed cells with either NRB-AF12 or ER-Tr, and antibodies directed against the Golgi structural protein GM130 (α-GM130). Data obtained, shown in Figure 5A, indicates a co-localization of NRB-AF12 and α-GM130 (Pearson's coefficient: 0.53 ± 0.02; *n* ≥ 10; Figure 5C). Similar results were obtained when α-GM130 was used in combination with ER-Tr (Pearson's coefficient: 0.58 ± 0.02; *n* ≥ 10; Figures 5B,C).

**Figure 6:**
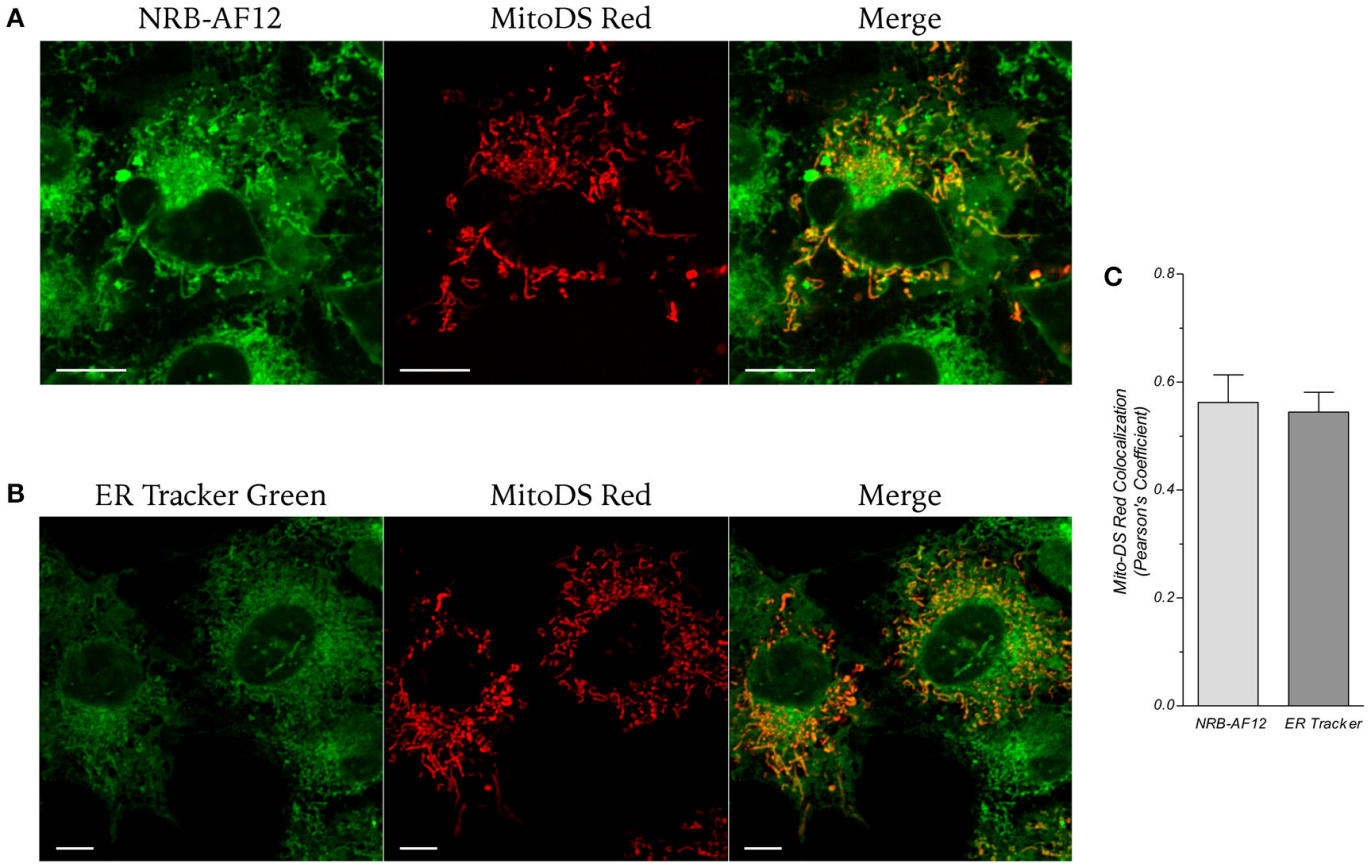
**Live cell imaging of liver myofibroblasts transfected with the mitochondrial marker Mito-Ds red and then stained using (A) NRB-AF12 or (B) ER-Tr green**. Magnification 60x; scale bar 10 μm. **(C)** Co-localization of NRB-AF12 and ER-Tr within mitochondria is reported as Pearson's coefficient (*n* ≥ 10).

Mitochondrial NRB-AF12 and ER-Tr localization was investigated in living LX2 cells transfected with the mitochondrial marker MitoDS-red plasmid, and then loaded with either NRB-AF12 or ER-Tr. As depicted in Figure 6, both dyes labeled mitochondria to similar levels (Pearson's Coefficient: 0.56 ± 0.05 and 0.54 ± 0.03, respectively for NRB-AF12 and ER-Tr; *n* ≥ 10).

**Figure 7:**
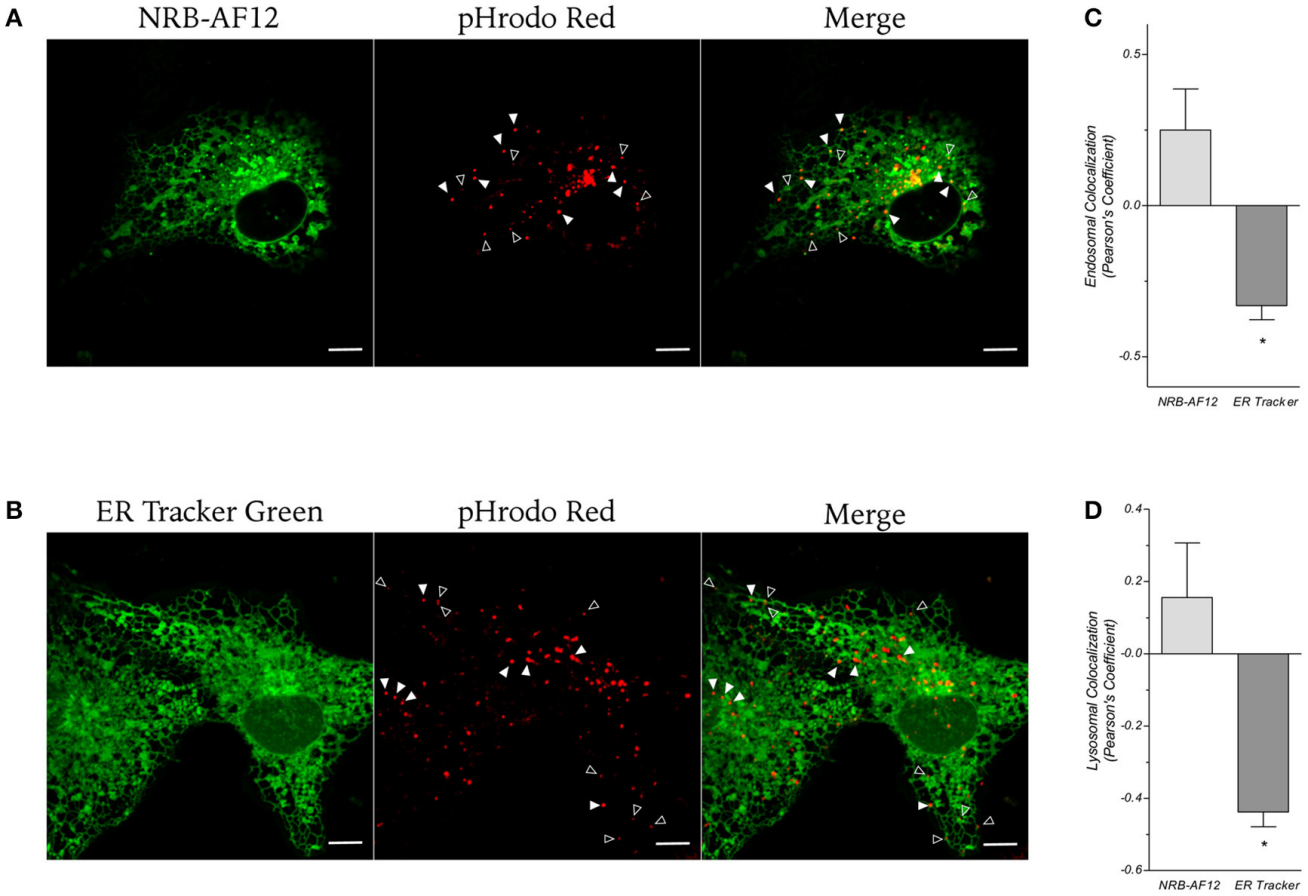
**Confocal live cell microphotographs of LX2 cells stained with the fluorescent dye for endosomal (empty arrowhead) and lysosomal (full arrowhead) compartments pHrodo red and (A) NRB-AF12 or (B) ER-Tr green**. Magnification 60x; scale bar 10 μm. **(C,D)** Co-localization of NRB-AF12 and ER-Tr Green with endosome and lysosome vesicles is shown as Pearson's coefficient (^*^p < 0.05; n ≥ 10).

Next, we investigated whether NRB-AF12 and ER-Tr could label endosomes and lysosomes. For this purpose, we performed live cell imaging experiments in LX2 cells using a commercial fluorescent probe which has been previously demonstrated to highlight vesicles involved in endocytosis. As shown in Figure 7, we could not find any staining of endocytic vesicles when cells were loaded with ER-Tr (Pearson's coefficient: −0.33 ± 0.05 and −0.43 ± 0.04, respectively for endosomes and lysosomes; *n* ≥ 10). In contrast, NRB-AF12 showed an appreciable co-localization rate with both endosomal and lysosomal vesicles (Pearson's coefficient 0.25 ± 0.13 and 0.15 ± 0.15, respectively; *n* ≥ 10). Interestingly, the data revealed a high index of dispersion of the co-localization coefficients (CI 95%: −0.05 – 0.55 and −0.19 – 0.5, for endosomes and lysosomes respectively; *n* ≥ 10), indicating that not all endosomes/lysosomes vesicles were stained by the dye.

**Figure 8:**
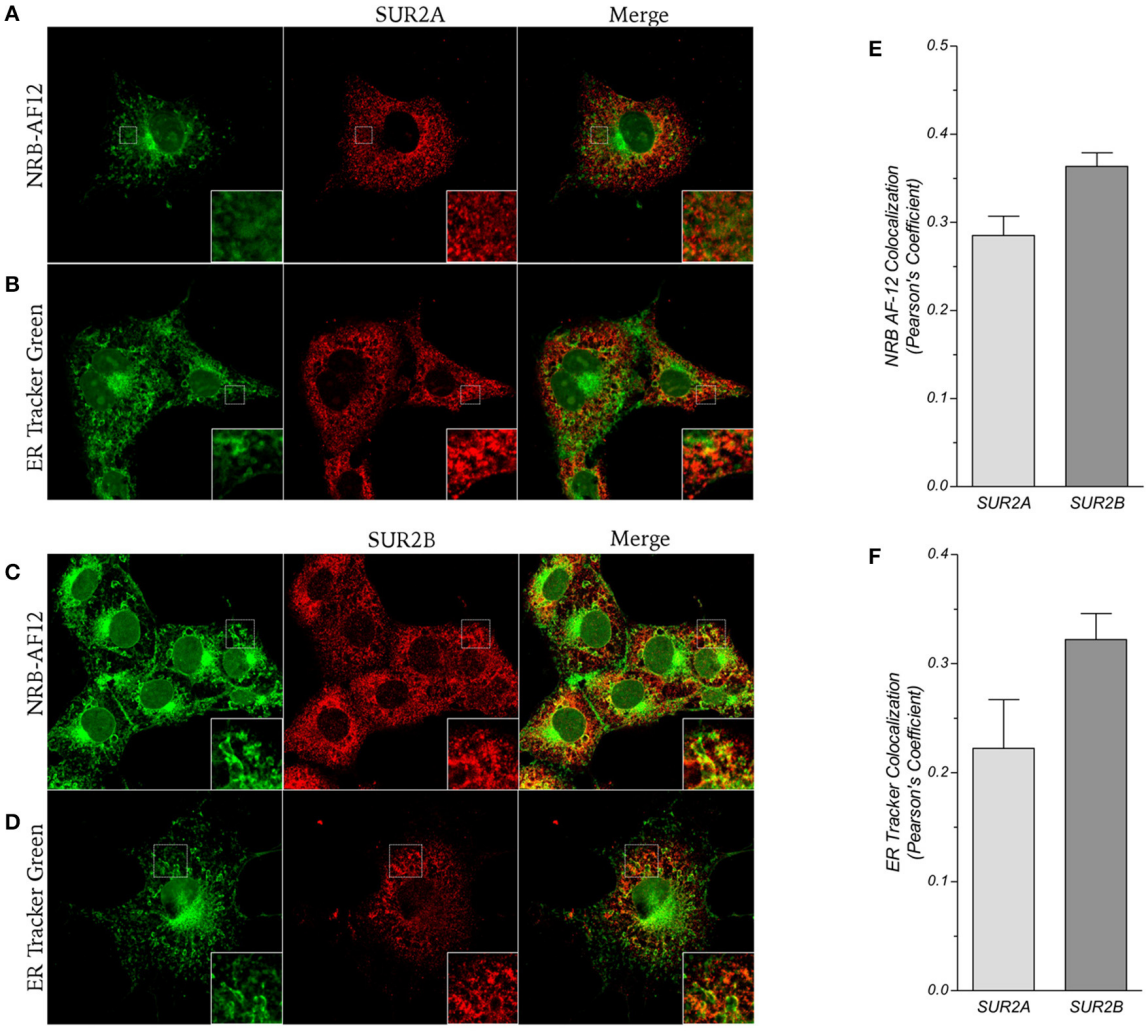
**(A,B)** Confocal pictures of LX2 fixed cells immunostained for the K_ATP_ channels subunit SUR2A and counter-labeled with **(A)** NRB-AF12 or **(B)** ER-Tr green. **(C,D)** Immunostaining of LX2 cells for SUR2B subunit of K_ATP_ channels; cells were counter-labeled with **(C)** NRB-AF12 or **(D)** ER-Tr green. Insets show magnification of the pictures. Magnification 60x; scale bar 10 μm. **(E,F)** Pearson's coefficient showing co-localization rate of **(E)** NRB-AF12 or **(F)** ER-Tr green with either SURs isoforms (n ≥ 15).

Since NRB-AF12 and ER-Tr showed the same cellular labeling pattern, we hypothesized a common target for these compounds. ER-Tr probes are conjugates of glibenclamide bearing different fluorophores, BODIPY FL (ER-Tr green) and BODIPY TR (ER-Tr red). Glibenclamide is an antidiabetic drug belonging to the class of sulfonylureas that binds the sulfonylurea receptor (SUR) subunits of ATP-sensitive potassium channels (Proks et al., 2002). Accordingly, we therefore explored the likelihood of whether NRB could also target these SURs by analyzing the co-localization between NRB-AF12 and the 2 major subunits SUR2A and SUR2B. We focused our attention on these SUR isoforms because of their intracellular distribution (Bao et al., 2011) and the role of the plasmalemmal SUR2B in the regulation of vascular contraction (Morrissey et al., 2005; Teramoto, 2006; Jackson, 2008; Tinker et al., 2014). Our results indicate that in LX2 cells NRB-AF12 co-localized with both SUR2A and SUR2B, with a greater correlation for the latter (Pearson's coefficient: 0.28 ± 0.02 and 0.36 ± 0.01, respectively for SUR2A and SUR2B; *n* ≥ 10) (Figures 8A,C,E); comparable results were also obtained with ER-Tr (Pearson's coefficient: 0.22 ± 0.04 and 0.32 ± 0.02, respectively for SUR2A and SUR2B; *n* ≥ 10) (Figure 8B,D,F). It is worth noting that the cellular distribution of NRB-AF12 and ER-Tr fluorescence was larger than that observed with SUR2, suggesting non-SUR2 additional targets for both dyes.

The overlapping fluorescence distributions of AF-12 and ER-Tr prompted us to investigate whether NRB and glibenclamide could compete with their respective fluorescent derivatives in LX2 cells. To this end, LX2 cells were pre-treated with high concentrations (100 μM) of either NRB or glibenclamide, and then loaded with either NRB-AF12 or ER-Tr. Interestingly, pre-treatment with the non-labeled compounds failed to prevent fluorescent labeling following addition of the fluorescent derivatives (data not shown).

To summarize the results concerning the cellular distribution of AF-12, we show in **Figure 10** a single living LX2 cell, in which are detailed the subcellular organelles labeled by the dye.

**Figure 9:**
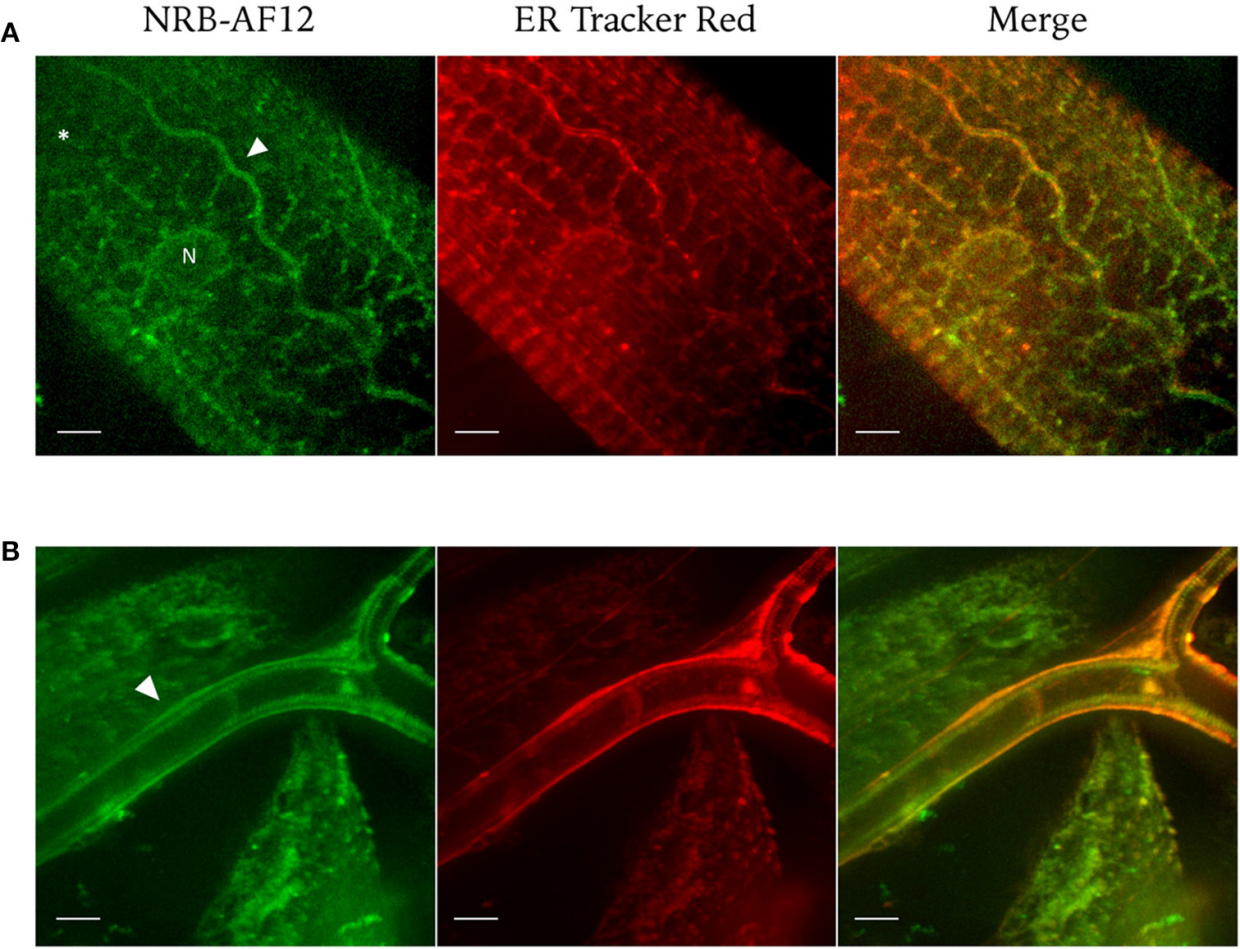
**Confocal live imaging of ***Drosophila*** (A) muscles and (B) tracheal system labeled with NRB-AF12 (green) and ER-Tr red (red)**. Both fluorescent dyes, added to the dissected larva in HL3 physiological solution, show an overlapping pattern of distribution as highlight by the yellow signal of the merged images (^*^: muscle; white arrowhead: trachea; N: nucleus). Magnification 60x; scale bar 10 μm.

We also explored the fluorescence features of NRB-AF12 in intact tissues of dissected larvae of *Drosophila melanogaster. Third* instar larvae were labeled with NRB-AF12 and ER-Tr dissolved in the hemolymph-like HL3 saline, added to the medium following dissection and imaged with an immersion objective. As shown in Figure 9, a bright fluorescence was observed when larvae were treated with the fluorescent probes: *In vivo* live imaging of *D. melanogaster* muscle fibers (Figure 9A) and tracheal system (Figure 9B) revealed a sarcoplasmic reticulum distribution of the dyes, with NRB-AF12 and ER-Tr fluorescence significantly overlapping, as was observed in cell culture (Johnson et al., 2015). Finally, we investigated some physical properties of NRB-AF-12 such as penetration, internalization rate, retention time, washout time-course in living cells, as well as the induction of cytotoxicity, in order to establish the usefulness and reliability of the dye. Video 1 in Supplementary Material, shows that in living LX2 cells AF-12 is internalized in minutes and is retained in the cells for at least 3 h following its removal from the culture medium. Furthermore, re-exposure of the cells to dye, gave the same results, in terms of fluorescence distribution, compared to the first exposure. NRB-AF12 was apparently not toxic in both human and rat cell lines, even at concentrations much higher than those (500 nM) utilized for labeling (**Figures 11B–D**).

## Discussion

This study was undertaken to verify if a fluorescent derivative of NRB could furnish information about the cellular target/s involved in the selective rat-specific and tissue-selective action of unlabeled NRB. To this end, we attached the NBD fluorophore to the NRB molecule. The NBD group is known to bleach very rapidly in respect to other commercially available fluorophores, nevertheless we decided to use NBD because of its relatively small size, in the attempt to minimize the risk of inducing a distortion of the chemical-physical properties of NRB. We compared the fluorescence distribution of NBD-NRB (NRB-AF12) observed in NRB-sensitive cells (freshly isolated rat caudal artery myocytes) with that observed in NRB-insensitive cells (several cultured cell lines). Preliminary experiments conducted in our lab indicated that NRB-AF12 has the same species- and tissue-specific contractile properties of unlabeled NRB (manuscript in preparation), and therefore is a reliable tool for the purpose of this work.

There are two key observations:

1. In both NRB-sensitive and NRB-insensitive cells NRB-AF12 distributes intracellularly; however, only in the sensitive cells is fluorescence detected in the plasma membrane.
2. In all the cells investigated, NRB-AF12 fluorescence showed a pattern of distribution similar to that of ER-Tr.

Given that the cellular labeling patterns of NRB-AF12 and ER-Tr are near identical, it could be proposed that the two dyes share a common cellular target/s. ER-Tr is a BODIPY derivative of glibenclamide, a widely used antidiabetic drug belonging to the sulfonylurea family. Glibenclamide is a non-selective inhibitor of ATP-sensitive potassium channels (Proks et al., 2002), a multimeric protein complex consisting of four pore-lining inward rectifier α subunits (K_ir_6.1/2) and four regulatory sulfonylurea-binding β subunits (SUR1/2A/2B) that belong to the ATP-binding cassette family (Yokoshiki et al., 1998; Zerangue et al., 1999; Shi et al., 2005; Ng et al., 2010). Co-assembly of different K_ir_ and SUR subunits generates ion channel combinations with different channel properties, and with different cell compartment or tissue distribution (Yokoshiki et al., 1998), such as the SUR1/K_ir_6.2 pancreatic type, the SUR2A/K_ir_6.2 cardiac type and the SUR2B/K_ir_6.1 vascular smooth muscle type. The ER-Tr and NRB-AF12 fluorescence characteristics may therefore reflect the cellular distribution of K_ATP_ channels. Inhibition of plasma membrane ATP-sensitive potassium channels leads to an increased intracellular K^+^ concentration, plasma membrane depolarization, opening of voltage-dependent calcium channels, and increased intracellular Ca^2+^ concentration (Ko et al., 2008) that, in vascular smooth muscle, triggers the contractile process. It has been reported that vascular smooth muscle plasma membrane selectively expresses SUR2B subunits of K_ATP_ channels (Morrissey et al., 2005; Teramoto, 2006; Tinker et al., 2014). These data, together with the finding (this study) that NRB-AF12 localizes to the plasma membrane only in NRB-sensitive cells, might explain the species- and tissue-selective activity of NRB, by hypothesizing an inhibitory effect of the drug on rat-specific SUR2B-containing K_ATP_ channels expressed on the plasma membrane of the rat peripheral artery myocytes. In contrast to this hypothesis is the fact that neither unlabeled NRB nor glibenclamide are able to displace NRB-AF12 or ER-Tr from their binding site, which presents the prospect of a different/additional site of action from/to K_ATP_ channels. It also raises the question as to whether the fluorescently labeled compounds have the same binding characteristics of the corresponding unlabeled compounds. However, the lack of displacement is in itself not sufficient to rule out the possibility that NRB-AF12 does bind to K_ATP_ channels, with there being at least two plausible explanations: (1) NRB-AF12 and NRB could bind to different sites within the SUR2B moiety, thus allowing the attachment of both compounds; (2) the fluorophore group of NRB-AF12 may have multiple binding sites preventing its displacement by unlabeled NRB. The existence of more than one binding site, at least for NRB-AF12, is supported by the fact that this dye, in contrast to ER-Tr, was able to label both endosomes and lysosomes. As with SUR2-containing channels, NRB-AF12 appears to be similarly targeted to the endosomal/lysosomal pathway, supporting the idea that this compound can be recycled by endocytosis as has been shown for the K_ATP_ channel subunits (Bao et al., 2011).

**Figure 10:**
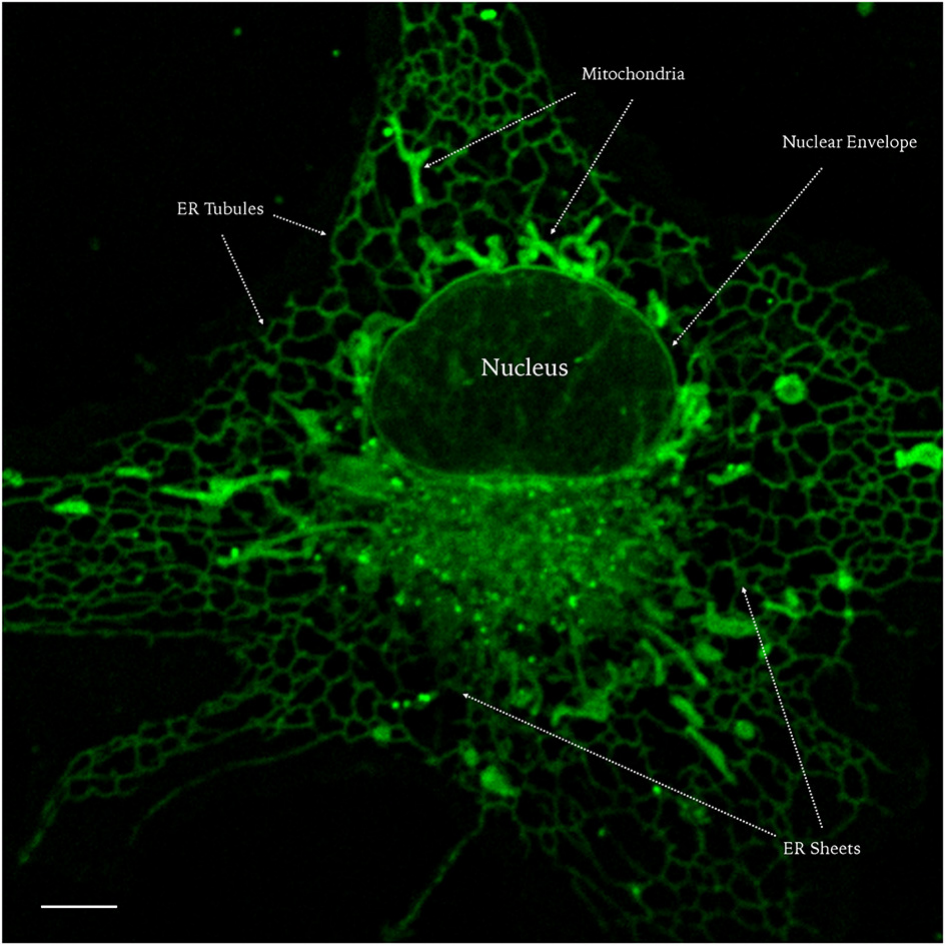
**Confocal live imaging of a LX2 cell stained with NRB-AF12 showing subcellular distribution of the fluorescent probe**. Magnification 100x; scale bar 5 μm.

**Figure 11:**
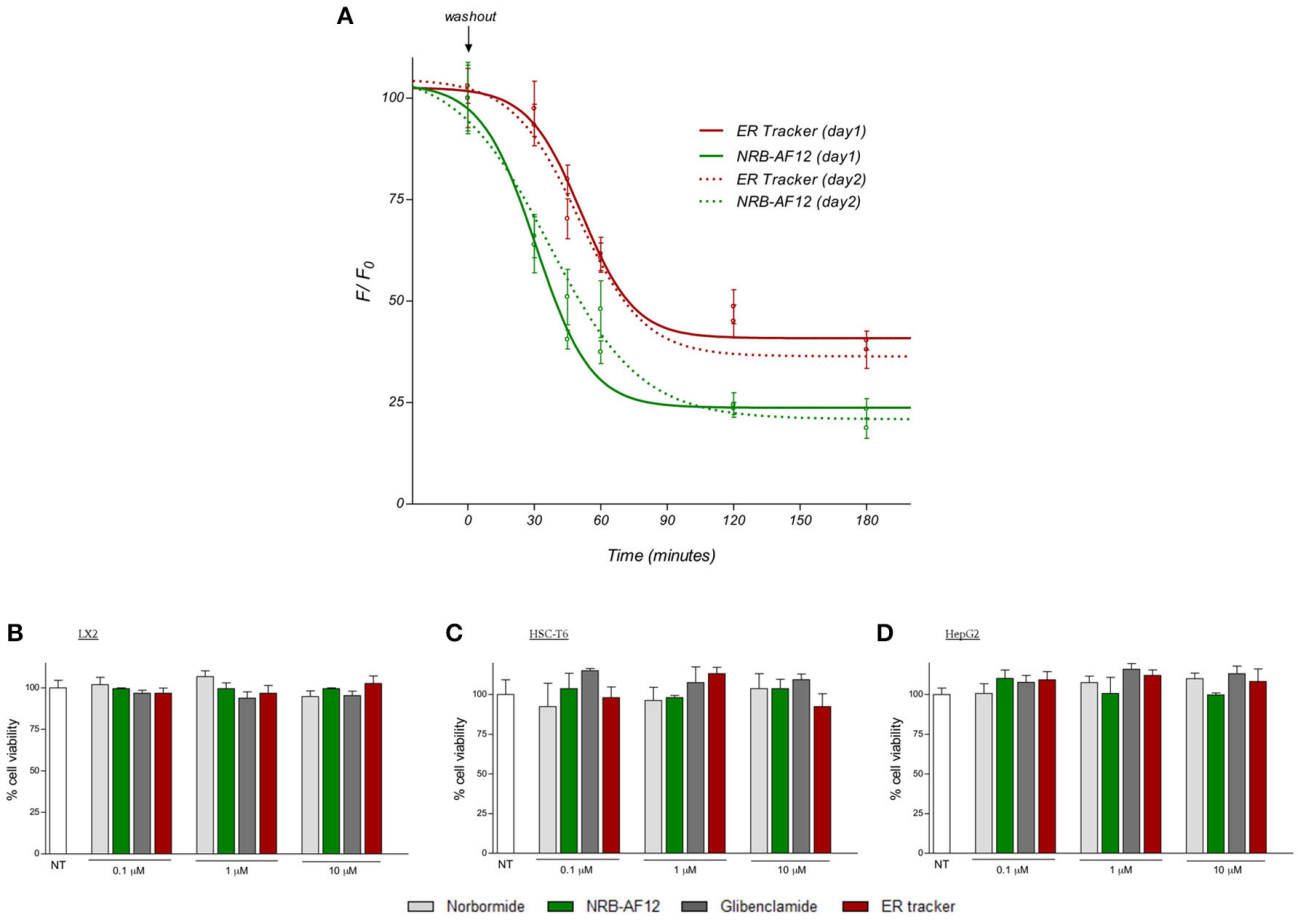
**(A)** LX2 cells were incubated for 30 min with NRB-AF12 or ER-Tr green; after replacing the staining solution with probe-free medium, evaluation of NRB-AF12 and ER-Tr extent of fluorescent signal and photostability was performed. **(B)** LX2, **(C)** HSC-T6, and **(D)** HepG2 cells were treated for 24 h with increasing doses (0.1, 1 and 10 μM) of NRB, NRB-AF12, glibenclamide or ER-Tr. Compounds toxicity was evaluated by trypan blue exclusion test of cell viability. NT, not treated cells.

Live cell imaging represents an important technique in the study of biological processes; the use of fluorescent proteins or dyes provides an important tool for the *in vivo* visualization, in space, and time, of virtually any cellular mechanism or structure, avoiding artifacts or sample alteration that could occur with fixation methods. In this study we show that NRB-AF12 is endowed with features that make this dye an eligible prototype of new fluorescent probes, alternatives to ER-Tr, for live cell imaging (Figure 10). Furthermore, in NRB-sensitive single rat vascular myocytes, this dye, due to its constriction effect (manuscript in preparation), could also be employed to study the morphologic changes that occur in labeled intracellular organelles, as well as in the plasma membrane, during the contractile process. Whatever the binding site/s for NRB-AF12, this dye shows a cellular distribution near identical to that of ER-Tr in real-time fluorescent imaging using different cultured cell lines and freshly isolated rat caudal artery myocytes. NRB-AF12 labeling was also observed in fixed and permeabilized cells, allowing its use in immunocytochemistry co-localization experiments. In all cells tested, NRB-AF12 stained not only ER, but also other subcellular compartments such as Golgi apparatus, mitochondria, and organelles involved in the endocytic pathway (i.e., endosomes and lysosomes). NRB-AF12 internalization rate was extremely fast, since cell incubation with the fluorescent compound resulted in a well-defined meshwork staining of the cellular structures within a few minutes, and could be observed up to 3 h following its removal from the culture medium (Figure 11A). Another useful feature is that NRB-AF12 exhibited no apparent cytotoxicity, a serious disadvantage that often occurs using fluorescent probes for live cell imaging. Cell viability experiments demonstrated the dye to be biocompatible, having no harmful effects in the different cell lines (both human and rat) after 24-h incubation, even at concentrations (10 μM) much higher than those (500 ηM) needed to visualize cellular organelles (Figures 11B–D). Furthermore, the lack of cytotoxicity of NRB-AF12 allows the same sample to be repeatedly stained with the dye (for example at different time points within an experiment), without morphological alteration of cell structures or cell death; concurrently, the ability of NRB to effectively label its cellular target is preserved on re-exposure to the dye (Figure 11A).

Finally, imaging experiments performed in dissected larvae of *D. melanogaster* demonstrated NRB-AF12 adaptability in complex biological systems. The live imaging of fly tissues showed that NRB-AF12 maintain the same subcellular distribution of ER-Tr also *in vivo*, supporting the theory of a common target. Moreover, we observed a significant signal in the *Drosophila* tracheal system where Drosophila Sur (Dsur) is highly expressed (Nasonkin et al., 1999). Dsur, encodes a glibenclamide-sensitive potassium channel and has been identified as the functional orthologue of the mammalian SUR2 (Kim and Rulifson, 2004). On the basis of these favorable properties, we propose NRB-AF12 as an alternative fluorescent dye to ER-Tr, and a new improved tool for labeling intracellular organelles.

In conclusion, this study presents evidence to demonstrate that NRB-AF12, a fluorescent derivative of the selective rat toxicant NRB, has a highly similar fluorescence distribution to ER-Tr, a fluorescent derivative of glibenclamide, a known K_ATP_ channel inhibitor. On the basis of these results we hypothesize common cellular binding sites for NRB and glibenclamide that may potentially lead to common biological properties. Furthermore, we propose NRB-AF12 as a prototype for the development of new fluorescent probes selective for intracellular structures/organelles. Studies are in progress in our lab to investigate these aspects.

## Author contributions

CD and GO contributed equally to this work. SB, CD, and MP conceived the study. AFe, MB, DR, BH, and GR designed and performed synthesis of NRB-AF12. CD, GO, GM, FF, AFo, GC, and SD designed and performed experiments. SB, CD, and GO analyzed and interpreted data. SB, CD, GO, DR, and BH wrote the manuscript. All authors approved the final version of the manuscript.

## Funding

This study was supported by the University of Padova, project n. 148125/14.

### Conflict of interest statement

The authors declare that the research was conducted in the absence of any commercial or financial relationships that could be construed as a potential conflict of interest.
