# Supplementary material for: An NBD Derivative of the Selective Rat Toxicant Norbormide as a New Probe for Living Cell Imaging
Source: Front Pharmacol. 2016 Sep 23;7:315. doi: 10.3389/fphar.2016.00315 (PMC5034647; doi:10.3389/fphar.2016.00315)
Supplement: Supplementary file 2 [file DataSheet1.DOCX]

**Supplementary material**

**Figure S1. Excitation and emission spectra of NRB-AF12.**

The compound was dissolved in 100% DMSO to obtain a 2 mM stock solution, then diluted in Milli-Q water to reach the final concentration.

**Description of movie 1**

Time-lapse recording of NRB-AF12 internalization in LX2 cells. Cells were exposed to NRB-AF12 (500 nM) and imaged every 10 seconds at a single focal plan for 5 minutes. Scale bar represents 10 μm.
