# Supplementary figures and images for: An NBD Derivative of the Selective Rat Toxicant Norbormide as a New Probe for Living Cell Imaging
Source: Front Pharmacol. 2016 Sep 23;7:315. doi: 10.3389/fphar.2016.00315 (PMC5034647; doi:10.3389/fphar.2016.00315)

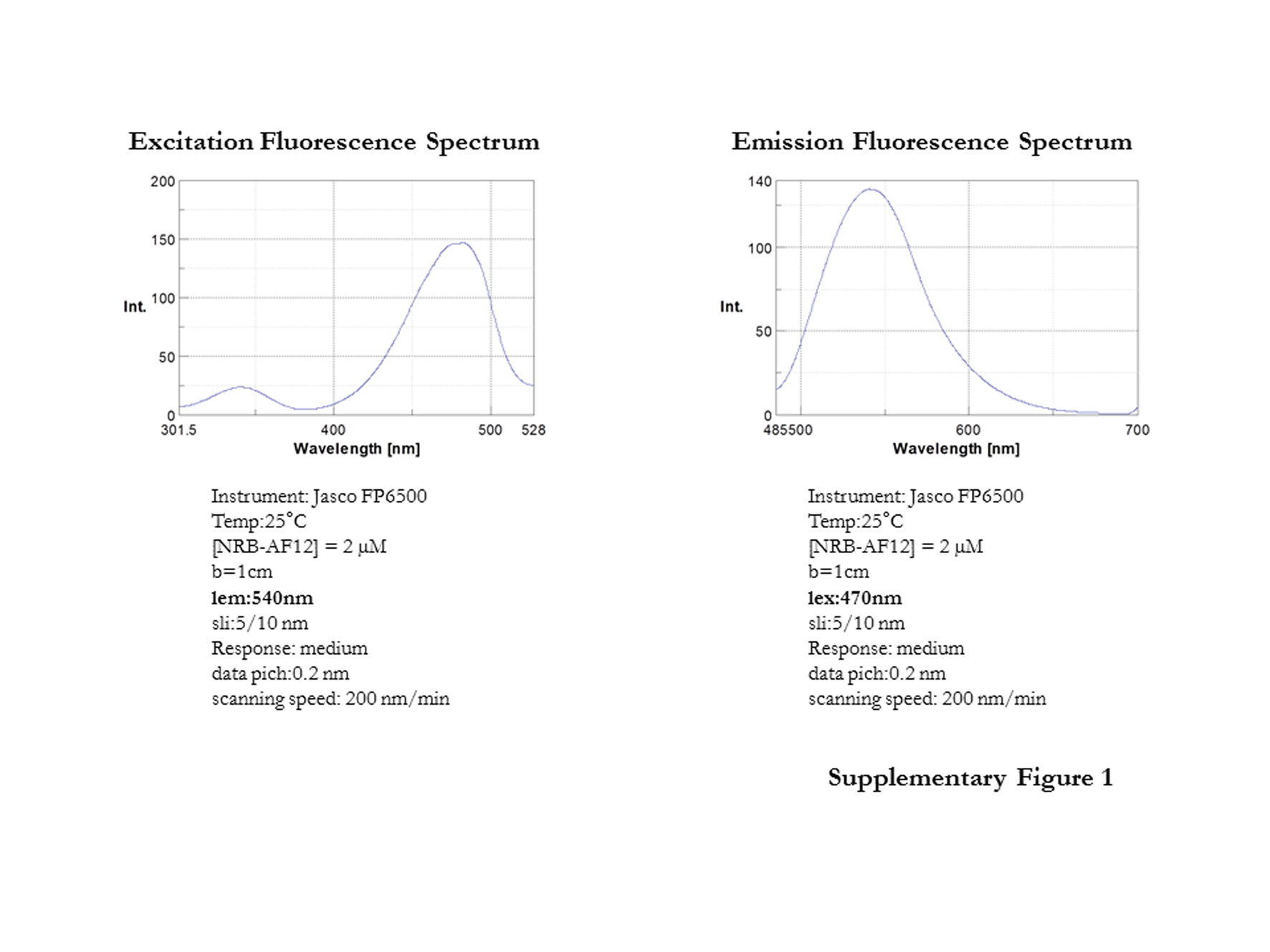

Supplement: Supplementary file 3 [file Image1.TIF]
